# *catena*-Poly[diquinolinium [[diaqua­cobaltate(II)]-μ-cyclo­tetra­phosphato] hexa­hydrate]

**DOI:** 10.1107/S1600536810002096

**Published:** 2010-01-23

**Authors:** Hanène Hemissi, Mohammed Rzaigui, Zeid Abdullah Al Othman

**Affiliations:** a Laboratoire de Chimie des Matériaux, Faculté des Sciences de Bizerte, 7021 Zarzouna Bizerte, Tunisia; b Chemistry Departement, Faculty of Sciences, King Saud University, Riyadh, Saudi Arabia

## Abstract

The cyclo­tetra­phosphate anion, [P_4_O_12_]^4−^, forms the title complex with cobalt(II) and quinolinium, {(C_9_H_8_N)_2_[Co(P_4_O_12_)(H_2_O)_2_]·6H_2_O}_*n*_. In the complex anion, the Co^II^ ion, lying on an inversion center, is surrounded by four phosphate O atoms and two water mol­ecules in a slightly distorted octa­hedral geometry. The crystal structure consists of anionic ribbons of formula {[Co(P_4_O_12_)(H_2_O)_2_]^2−^}_*n*_ extending along [100]. A network of O—H⋯O, N—H⋯O and C—H⋯O hydrogen bonds consolidates the crystal packing.

## Related literature

For the crystal chemistry of condensed phosphates, see: Durif (1995 ▶). For general background to transition metal–organic derivatives of polyoxoanions, see: Feher & Budzichowski (1995 ▶); Guerrero *et al.* (1999 ▶); Ikotun *et al.* (2008 ▶); Lugmair & Tilley (1998 ▶). For general background to hydrogen bonds, see: Blessing (1986 ▶); Brown (1976 ▶); Steiner & Saenger (1993 ▶). For the synthesis, see: Ondik (1964 ▶).
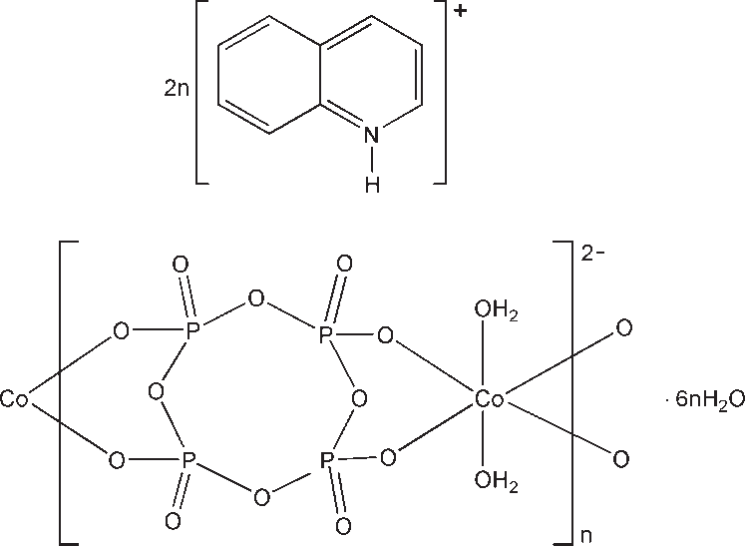


## Experimental

### 

#### Crystal data

(C_9_H_8_N)_2_[Co(P_4_O_12_)(H_2_O)_2_]·6H_2_O
*M* *_r_* = 779.27
Triclinic, 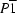

*a* = 7.443 (3) Å
*b* = 10.037 (4) Å
*c* = 10.682 (7) Å
α = 83.74 (4)°
β = 70.98 (4)°
γ = 85.71 (3)°
*V* = 749.4 (6) Å^3^
*Z* = 1
Ag *K*α radiation
λ = 0.56083 Å
μ = 0.46 mm^−1^
*T* = 293 K
0.20 × 0.18 × 0.16 mm

#### Data collection

Enraf–Nonius TurboCAD-4 diffractometer
12878 measured reflections
7242 independent reflections
4531 reflections with *I* > 2σ(*I*)
*R*_int_ = 0.039
2 standard reflections every 120 min  intensity decay: 2%

#### Refinement

*R*[*F* ^2^ > 2σ(*F* ^2^)] = 0.057
*wR*(*F* ^2^) = 0.164
*S* = 0.98
7242 reflections
237 parameters
13 restraints
H atoms treated by a mixture of independent and constrained refinement
Δρ_max_ = 1.11 e Å^−3^
Δρ_min_ = −1.46 e Å^−3^

### 

Data collection: *CAD-4 EXPRESS* (Enraf–Nonius, 1989 ▶); cell refinement: *CAD-4 EXPRESS*; data reduction: *XCAD4* (Harms & Wocadlo, 1995 ▶); program(s) used to solve structure: *SHELXS97* (Sheldrick, 2008 ▶); program(s) used to refine structure: *SHELXL97* (Sheldrick, 2008 ▶); molecular graphics: *ORTEP-3* (Farrugia, 1997 ▶); software used to prepare material for publication: *WinGX* (Farrugia, 1999 ▶).

## Supplementary Material

Crystal structure: contains datablocks I, global. DOI: 10.1107/S1600536810002096/hy2272sup1.cif

Structure factors: contains datablocks I. DOI: 10.1107/S1600536810002096/hy2272Isup2.hkl

Additional supplementary materials:  crystallographic information; 3D view; checkCIF report

## Figures and Tables

**Table 1:** Hydrogen-bond geometry (Å, °)

*D*—H⋯*A*	*D*—H	H⋯*A*	*D*⋯*A*	*D*—H⋯*A*
N1—H1⋯O2	0.86	1.88	2.728 (3)	171
O1—H11⋯O4	0.83 (3)	1.98 (3)	2.747 (3)	154 (3)
O1—H21⋯O2^i^	0.84 (3)	2.00 (3)	2.841 (3)	179 (4)
O2—H12⋯O3^ii^	0.83 (3)	1.85 (3)	2.666 (3)	168 (3)
O2—H22⋯O6	0.83 (3)	1.95 (3)	2.771 (3)	172 (4)
O3—H13⋯O6	0.86 (2)	1.95 (2)	2.743 (3)	152 (3)
O3—H23⋯O5^iii^	0.87 (3)	2.02 (2)	2.833 (3)	154 (3)
O4—H14⋯O9^iv^	0.86 (2)	2.01 (2)	2.824 (3)	157 (4)
O4—H24⋯O9^i^	0.86 (3)	2.06 (3)	2.890 (3)	163 (3)
C7—H7⋯O9^v^	0.93	2.59	3.459 (4)	156
C9—H9⋯O3	0.93	2.54	3.080 (4)	118
C9—H9⋯O10^vi^	0.93	2.59	3.381 (3)	143

Symmetry codes: (i) 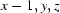; (ii) 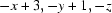; (iii) 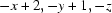; (iv) 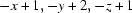; (v) 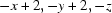; (vi) 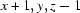.

**Fig. 1.**
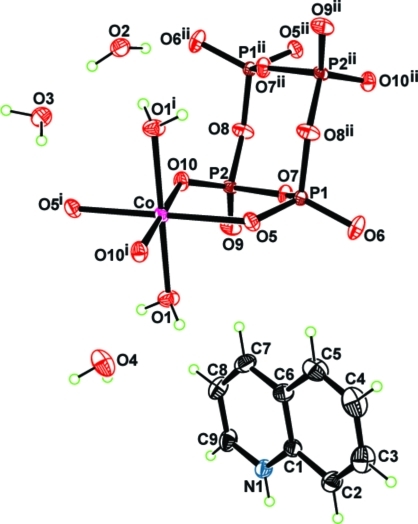
The structure of the title compound. Displacement ellipsoids are drawn at the 30% probability level. [Symmetry codes: (i) 1-x, 1-y, 1-z; (ii) 2-x, 1-y, 1-z.]

**Fig. 2.**
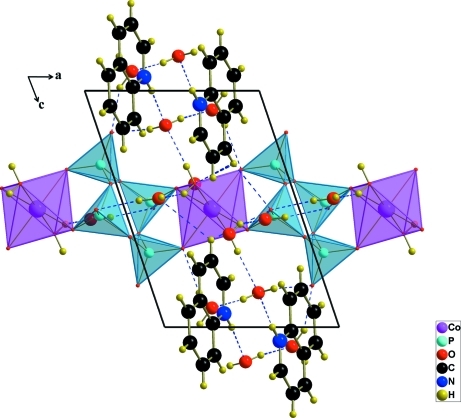
Projection of the title compound along the b axis.

## supplementary crystallographic information

### Comment

Transition metal–organic derivatives of polyoxoanions have recently been attracting considerable attention, because they serve as molecular models of metal species bound on oxo surfaces of heterogeneous catalysts (Feher & Budzichowski, 1995). In this context, several frameworks of this kind of materials with one-, two- or three-dimensional networks have been successfully constructed by using monophosphates, monophosphonates and monophosphinates (Guerrero *et al.*, 1999; Lugmair & Tilley, 1998). In contrast, structural diversity of transition metal–organic derivatives of condensed phosphates has been much less explored. In order to enrich the varieties in such kinds of hybrid materials, we report the synthesis and crystal structure of (C_9_H_8_N)_2_[Co(P_4_O_12_)(H_2_O)_2_].6H_2_O.

The title compound contains protonated quinolinium cations, diaquacyclotetraphosphatocobaltate(II) dianions and water molecules (Fig. 1). The cyclic phosphate anion, [P_4_O_12_]^4-^, is located around an inversion center and so is built up by only two independent PO_4_ tetrahedra. Its main geometrical features [the bond lengths P—O = 1.473 (2)–1.603 (2) Å, and the bond angles O—P—O = 100.24 (9)–121.11 (1)°, P—O—P = 134.40 (1)–137.25 (1)°] are not significantly different from what is commonly observed in other cyclotetraphosphate anions with the same internal symmetry (Durif, 1995). The coordination polyhedron of the Co^II^ atom, which lies on an inversion center, is octahedral with four external O atoms O(E) from two adjacent bidentate cyclotetraphosphates and two water O atoms O(w), providing a Co atom with six O donor set [four O(E) equatorial arrangement with two axial O(w)]. The Co—O bond lengths fall within the range of 2.106 (2)–2.116 (2) Å. The shortest distance between two neighboring Co atoms is 7.443 (3) Å. This distance could explain the cobalt magnetic properties in several materials (Ikotun *et al.*, 2008). The [CoO_4_(H_2_O)_2_] octahedra alternate with the P_4_O_12_ rings as to form infinite ribbons, fused through Co—O—P linkage, propagating along the *a* axis (Fig. 2). The protonated quinolinim is located in the inter-ribbons spacing, and it neutralizes the negative charge of the anionic part. These organic entities are planar as evidenced by the mean deviation (±0.005 Å) from least square plane defined by the nine constituent atoms. As well as electrostatic and van der Waals interactions, the component species of the title compound establish a three-dimensional network through N—H···O and O—H···O hydrogen bonds. The structure is further stabilized with non-classical hydrogen bonds of the C—H···O type (Steiner & Saenger, 1993). The examination of the hydrogen-bond scheme shows that hydrogen bond connecting C9 to the phosphate group and water molecule is bifurcated. In the structure, there are two strong hydrogen bonds, with O···O distances of 2.666 (3) and 2.728 (3) Å. The others are weaker, with O(N, C)···O ranging from 2.743 (3) to 3.459 (4) Å (Blessing, 1986; Brown, 1976).

### Experimental

The title compound was prepared by adding ethanolic solution (5 ml) of quinoline (8.34 mmol) dropwise to a mixture of H_4_P_4_O_12_ (4.15 mmol) and CoCl_2_ (4.15 mmol) in water (20 ml). Pink prism crystals of good quality were obtained after a slow evaporation during few days at ambient temperature. The cyclotetraphosphoric acid, H_4_P_4_O_12_, was produced from Na_4_P_4_O_12_.4H_2_O, prepared according to the Ondik process (Ondik, 1964) through an ion-exchange resin in H-state (Amberlite IR 120).

### Refinement

H atoms on C and N atoms were positioned geometrically and treated as riding on their parent atoms, with N—H = 0.86, C—H =0.93 Å and with *U*_iso_(H) = 1.2*U*_eq_(C,N). H atoms of water molecules were located from difference Fourier maps and refined isotropically. The highest residual electron density was found 0.73 Å from Co1 and the deepest hole 0.76 Å from Co1.

### Crystal data

**Table:**

(C_9_H_8_N)_2_[Co(P_4_O_12_)(H_2_O)_2_]·6H_2_O	*Z* = 1
*M**_r_* = 779.27	*F*(000) = 401
Triclinic, *P*1	*D*_x_ = 1.727 Mg m^−^^3^
Hall symbol: -P 1	Ag *K*α radiation, λ = 0.56083 Å
*a* = 7.443 (3) Å	Cell parameters from 25 reflections
*b* = 10.037 (4) Å	θ = 9.0–11.0°
*c* = 10.682 (7) Å	µ = 0.46 mm^−^^1^
α = 83.74 (4)°	*T* = 293 K
β = 70.98 (4)°	Prism, pink
γ = 85.71 (3)°	0.20 × 0.18 × 0.16 mm
*V* = 749.4 (6) Å^3^	

### Data collection

**Table:**

Enraf–Nonius TurboCAD-4 diffractometer	*R*_int_ = 0.039
Radiation source: fine-focus sealed tube	θ_max_ = 28.0°, θ_min_ = 2.2°
graphite	*h* = −12→12
non–profiled ω/2θ scans	*k* = −16→16
12878 measured reflections	*l* = −17→10
7242 independent reflections	2 standard reflections every 120 min
4531 reflections with *I* > 2σ(*I*)	intensity decay: 2%

### Refinement

**Table:**

Refinement on *F*^2^	Primary atom site location: structure-invariant direct methods
Least-squares matrix: full	Secondary atom site location: difference Fourier map
*R*[*F*^2^ > 2σ(*F*^2^)] = 0.057	Hydrogen site location: inferred from neighbouring sites
*wR*(*F*^2^) = 0.164	H atoms treated by a mixture of independent and constrained refinement
*S* = 0.98	*w* = 1/[σ^2^(*F*_o_^2^) + (0.097*P*)^2^] where *P* = (*F*_o_^2^ + 2*F*_c_^2^)/3
7242 reflections	(Δ/σ)_max_ = 0.022
237 parameters	Δρ_max_ = 1.11 e Å^−^^3^
13 restraints	Δρ_min_ = −1.46 e Å^−^^3^

### Fractional atomic coordinates and isotropic or equivalent isotropic displacement parameters (Å^2^)

**Table:**

	*x*	*y*	*z*	*U*_iso_*/*U*_eq_	
Co1	0.5000	0.5000	0.5000	0.02117 (10)	
P2	0.84035 (7)	0.67186 (5)	0.52866 (5)	0.02037 (11)	
P1	0.94639 (7)	0.49127 (5)	0.31420 (5)	0.02082 (11)	
O5	0.7442 (2)	0.47075 (18)	0.33361 (17)	0.0290 (3)	
O9	0.8351 (3)	0.81917 (16)	0.5033 (2)	0.0353 (4)	
O8	0.9759 (3)	0.63833 (17)	0.6180 (2)	0.0329 (4)	
O7	0.9688 (2)	0.61035 (16)	0.39418 (17)	0.0284 (3)	
O1	0.4503 (3)	0.68127 (18)	0.39353 (19)	0.0348 (4)	
O6	1.0732 (3)	0.5182 (2)	0.17668 (18)	0.0410 (4)	
C1	1.2832 (4)	0.9562 (2)	−0.0159 (3)	0.0314 (4)	
N1	1.3663 (3)	0.8353 (2)	−0.0577 (2)	0.0350 (4)	
H1	1.3836	0.7736	0.0002	0.042*	
C2	1.2288 (5)	0.9765 (3)	0.1194 (3)	0.0432 (6)	
H2	1.2488	0.9092	0.1811	0.052*	
C6	1.2539 (4)	1.0555 (3)	−0.1110 (3)	0.0370 (5)	
C7	1.3093 (5)	1.0269 (3)	−0.2441 (3)	0.0447 (6)	
H7	1.2879	1.0908	−0.3082	0.054*	
C3	1.1460 (6)	1.0973 (4)	0.1583 (4)	0.0549 (8)	
H3	1.1094	1.1128	0.2476	0.066*	
C8	1.3942 (5)	0.9057 (4)	−0.2799 (3)	0.0495 (7)	
H8	1.4340	0.8876	−0.3686	0.059*	
C9	1.4211 (4)	0.8100 (3)	−0.1843 (3)	0.0437 (6)	
H9	1.4780	0.7269	−0.2086	0.052*	
C5	1.1660 (5)	1.1797 (3)	−0.0655 (4)	0.0512 (8)	
H5	1.1433	1.2478	−0.1255	0.061*	
C4	1.1153 (6)	1.1986 (3)	0.0652 (4)	0.0607 (10)	
H4	1.0590	1.2805	0.0940	0.073*	
O10	0.6636 (2)	0.59823 (17)	0.58700 (16)	0.0282 (3)	
O2	1.3962 (3)	0.65569 (19)	0.14615 (19)	0.0358 (4)	
O3	1.3081 (3)	0.5195 (2)	−0.0816 (2)	0.0412 (4)	
O4	0.2153 (3)	0.9054 (2)	0.4559 (3)	0.0549 (6)	
H11	0.369 (4)	0.735 (3)	0.436 (3)	0.044 (10)*	
H21	0.436 (5)	0.674 (4)	0.320 (2)	0.052 (11)*	
H12	1.496 (3)	0.610 (3)	0.118 (4)	0.052 (11)*	
H22	1.299 (3)	0.613 (3)	0.163 (4)	0.046 (10)*	
H13	1.208 (4)	0.513 (4)	−0.013 (2)	0.052 (11)*	
H23	1.264 (5)	0.505 (4)	−0.145 (2)	0.056 (11)*	
H14	0.231 (5)	0.990 (2)	0.451 (4)	0.056 (11)*	
H24	0.098 (3)	0.897 (3)	0.465 (4)	0.056 (11)*	

### Atomic displacement parameters (Å^2^)

**Table:**

	*U*^11^	*U*^22^	*U*^33^	*U*^12^	*U*^13^	*U*^23^
Co1	0.01923 (17)	0.02359 (18)	0.02047 (18)	0.00158 (13)	−0.00587 (13)	−0.00424 (13)
P2	0.0235 (2)	0.0164 (2)	0.0224 (2)	0.00486 (16)	−0.00927 (18)	−0.00462 (16)
P1	0.0217 (2)	0.0241 (2)	0.0155 (2)	0.00357 (17)	−0.00460 (16)	−0.00431 (17)
O5	0.0228 (7)	0.0389 (9)	0.0272 (8)	0.0059 (6)	−0.0095 (6)	−0.0111 (6)
O9	0.0451 (10)	0.0176 (7)	0.0457 (11)	0.0086 (6)	−0.0190 (8)	−0.0054 (7)
O8	0.0385 (9)	0.0262 (7)	0.0439 (10)	0.0039 (6)	−0.0270 (8)	−0.0061 (7)
O7	0.0277 (7)	0.0257 (7)	0.0277 (8)	−0.0016 (6)	−0.0009 (6)	−0.0091 (6)
O1	0.0473 (10)	0.0289 (8)	0.0294 (9)	0.0092 (7)	−0.0153 (8)	−0.0057 (7)
O6	0.0442 (10)	0.0516 (11)	0.0189 (7)	−0.0067 (9)	0.0032 (7)	−0.0060 (7)
C1	0.0319 (10)	0.0283 (10)	0.0328 (12)	0.0007 (8)	−0.0094 (9)	−0.0019 (8)
N1	0.0377 (11)	0.0283 (9)	0.0329 (11)	0.0020 (8)	−0.0050 (8)	0.0013 (8)
C2	0.0487 (15)	0.0478 (15)	0.0337 (13)	0.0005 (12)	−0.0135 (11)	−0.0072 (11)
C6	0.0421 (13)	0.0286 (11)	0.0394 (13)	−0.0012 (9)	−0.0137 (11)	0.0025 (9)
C7	0.0512 (16)	0.0462 (15)	0.0356 (14)	−0.0076 (12)	−0.0154 (12)	0.0092 (12)
C3	0.064 (2)	0.0542 (19)	0.0495 (18)	−0.0019 (16)	−0.0158 (16)	−0.0233 (15)
C8	0.0562 (18)	0.0559 (18)	0.0300 (13)	−0.0067 (14)	−0.0044 (12)	−0.0033 (12)
C9	0.0460 (15)	0.0368 (13)	0.0383 (14)	0.0024 (11)	0.0004 (11)	−0.0071 (11)
C5	0.0574 (19)	0.0280 (12)	0.069 (2)	0.0018 (12)	−0.0237 (16)	−0.0006 (13)
C4	0.062 (2)	0.0364 (15)	0.082 (3)	0.0054 (14)	−0.0164 (19)	−0.0267 (17)
O10	0.0251 (7)	0.0347 (8)	0.0249 (7)	−0.0022 (6)	−0.0056 (6)	−0.0095 (6)
O2	0.0398 (10)	0.0339 (9)	0.0312 (9)	0.0008 (7)	−0.0094 (8)	0.0001 (7)
O3	0.0451 (11)	0.0543 (12)	0.0253 (9)	0.0030 (9)	−0.0129 (8)	−0.0072 (8)
O4	0.0449 (12)	0.0263 (9)	0.091 (2)	0.0085 (8)	−0.0178 (12)	−0.0164 (11)

### Geometric parameters (Å, °)

**Table:**

Co1—O10^i^	2.1064 (16)	N1—H1	0.8600
Co1—O10	2.1064 (16)	C2—C3	1.361 (5)
Co1—O1^i^	2.1127 (18)	C2—H2	0.9300
Co1—O1	2.1127 (18)	C6—C7	1.402 (4)
Co1—O5^i^	2.1159 (16)	C6—C5	1.421 (4)
Co1—O5	2.1159 (16)	C7—C8	1.362 (5)
P2—O9	1.4737 (17)	C7—H7	0.9300
P2—O10	1.4748 (17)	C3—C4	1.403 (6)
P2—O8	1.5963 (17)	C3—H3	0.9300
P2—O7	1.6029 (16)	C8—C9	1.377 (5)
P1—O6	1.4730 (18)	C8—H8	0.9300
P1—O5	1.4779 (17)	C9—H9	0.9300
P1—O8^ii^	1.5833 (18)	C5—C4	1.354 (6)
P1—O7	1.5913 (17)	C5—H5	0.9300
O8—P1^ii^	1.5833 (18)	C4—H4	0.9300
O1—H11	0.830 (17)	O2—H12	0.827 (17)
O1—H21	0.831 (17)	O2—H22	0.825 (17)
C1—N1	1.372 (3)	O3—H13	0.863 (17)
C1—C6	1.401 (4)	O3—H23	0.875 (17)
C1—C2	1.402 (4)	O4—H14	0.855 (18)
N1—C9	1.326 (4)	O4—H24	0.855 (17)
			
O10^i^—Co1—O10	180.00 (8)	N1—C1—C2	119.6 (2)
O10^i^—Co1—O1^i^	90.99 (7)	C6—C1—C2	122.1 (3)
O10—Co1—O1^i^	89.01 (7)	C9—N1—C1	122.4 (2)
O10^i^—Co1—O1	89.01 (7)	C9—N1—H1	118.8
O10—Co1—O1	90.99 (7)	C1—N1—H1	118.8
O1^i^—Co1—O1	180.0	C3—C2—C1	118.3 (3)
O10^i^—Co1—O5^i^	89.95 (6)	C3—C2—H2	120.8
O10—Co1—O5^i^	90.05 (6)	C1—C2—H2	120.8
O1^i^—Co1—O5^i^	86.22 (7)	C1—C6—C7	118.8 (3)
O1—Co1—O5^i^	93.78 (7)	C1—C6—C5	117.5 (3)
O10^i^—Co1—O5	90.05 (6)	C7—C6—C5	123.7 (3)
O10—Co1—O5	89.95 (6)	C8—C7—C6	120.1 (3)
O1^i^—Co1—O5	93.78 (7)	C8—C7—H7	119.9
O1—Co1—O5	86.22 (7)	C6—C7—H7	119.9
O5^i^—Co1—O5	180.0	C2—C3—C4	120.8 (3)
O9—P2—O10	121.11 (11)	C2—C3—H3	119.6
O9—P2—O8	105.29 (10)	C4—C3—H3	119.6
O10—P2—O8	110.13 (10)	C7—C8—C9	119.8 (3)
O9—P2—O7	108.09 (10)	C7—C8—H8	120.1
O10—P2—O7	109.91 (9)	C9—C8—H8	120.1
O8—P2—O7	100.23 (10)	N1—C9—C8	120.5 (3)
O6—P1—O5	117.41 (11)	N1—C9—H9	119.7
O6—P1—O8^ii^	109.43 (12)	C8—C9—H9	119.7
O5—P1—O8^ii^	106.81 (10)	C4—C5—C6	119.9 (3)
O6—P1—O7	106.60 (11)	C4—C5—H5	120.0
O5—P1—O7	111.38 (9)	C6—C5—H5	120.0
O8^ii^—P1—O7	104.47 (10)	C5—C4—C3	121.3 (3)
P1—O5—Co1	130.02 (10)	C5—C4—H4	119.3
P1^ii^—O8—P2	137.26 (12)	C3—C4—H4	119.3
P1—O7—P2	134.40 (11)	P2—O10—Co1	131.90 (10)
Co1—O1—H11	117 (2)	H12—O2—H22	113 (3)
Co1—O1—H21	116 (3)	H13—O3—H23	102 (2)
H11—O1—H21	110 (2)	H14—O4—H24	107 (3)
N1—C1—C6	118.3 (2)		

Symmetry codes: (i) −*x*+1, −*y*+1, −*z*+1; (ii) −*x*+2, −*y*+1, −*z*+1.

### Hydrogen-bond geometry (Å, °)

**Table:**

*D*—H···*A*	*D*—H	H···*A*	*D*···*A*	*D*—H···*A*
N1—H1···O2	0.86	1.88	2.728 (3)	171
O1—H11···O4	0.83 (3)	1.98 (3)	2.747 (3)	154 (3)
O1—H21···O2^iii^	0.84 (3)	2.00 (3)	2.841 (3)	179 (4)
O2—H12···O3^iv^	0.83 (3)	1.85 (3)	2.666 (3)	168 (3)
O2—H22···O6	0.83 (3)	1.95 (3)	2.771 (3)	172 (4)
O3—H13···O6	0.86 (2)	1.95 (2)	2.743 (3)	152 (3)
O3—H23···O5^v^	0.87 (3)	2.02 (2)	2.833 (3)	154 (3)
O4—H14···O9^vi^	0.86 (2)	2.01 (2)	2.824 (3)	157 (4)
O4—H24···O9^iii^	0.86 (3)	2.06 (3)	2.890 (3)	163 (3)
C7—H7···O9^vii^	0.93	2.59	3.459 (4)	156
C9—H9···O3	0.93	2.54	3.080 (4)	118
C9—H9···O10^viii^	0.93	2.59	3.381 (3)	143

Symmetry codes: (iii) *x*−1, *y*, *z*; (iv) −*x*+3, −*y*+1, −*z*; (v) −*x*+2, −*y*+1, −*z*; (vi) −*x*+1, −*y*+2, −*z*+1; (vii) −*x*+2, −*y*+2, −*z*; (viii) *x*+1, *y*, *z*−1.
